# Plasma Concentrations of Soluble Endoglin versus Standard Evaluation in Patients with Suspected Preeclampsia

**DOI:** 10.1371/journal.pone.0048259

**Published:** 2012-10-26

**Authors:** Sarosh Rana, Ana Sofia Cerdeira, Julia Wenger, Saira Salahuddin, Kee-Hak Lim, Steven J. Ralston, Ravi I. Thadhani, S. Ananth Karumanchi

**Affiliations:** 1 Division of Maternal Fetal Medicine, Beth Israel Deaconess Medical Center and Harvard Medical School, Boston, Massachusetts, United States of America; 2 Department of Obstetrics and Gynecology, Beth Israel Deaconess Medical Center and Harvard Medical School, Boston, Massachusetts, United States of America; 3 Department of Medicine, Beth Israel Deaconess Medical Center and Harvard Medical School, Boston, Massachusetts, United States of America; 4 Gulbenkian Programme for Advanced Medical Education, Lisbon, Portugal; 5 Division of Nephrology/Department of Medicine, Massachusetts General Hospital and Harvard Medical School, Boston, Massachusetts, United States of America; 6 Boston Maternal Fetal Medicine Group, Boston, Massachusetts, United States of America; 7 Howard Hughes Medical Institute, Chevy Chase, Maryland, United States of America; VU University Medical Center, The Netherlands

## Abstract

**Background:**

The purpose of this study was to compare plasma soluble endoglin (sEng) levels with standard clinical evaluation or plasma levels of other angiogenic proteins [soluble fms-like tyrosine kinase 1 (sFlt1) and placental growth factor (PlGF)] in predicting short-term adverse maternal and perinatal outcomes in women with suspected preeclampsia presenting prior to 34 weeks.

**Methods and Findings:**

Data from all women presenting at <34 weeks for evaluation of preeclampsia with singleton pregnancies (July 2009−October 2010) were included in this analysis and sEng levels were measured at presentation. Data was analyzed for 170 triage encounters and presented as median {25−75^th^ centile}. Thirty-three percent of patients (56 of 170) experienced an adverse outcome. sEng levels (ng/ml) were significantly elevated in patients who subsequently experienced adverse outcomes compared to those who did not (32.3 {18.1, 55.8} vs 4.8 {3.2, 8.6}, p<0.0001). At a 10% false positive rate, sEng had higher detection rates of adverse outcomes than the combination of highest systolic blood pressure, proteinuria and abnormal laboratory tests (80.4 {70.0, 90.8} vs 63.8 {51.4, 76.2}, respectively). Subjects in the highest quartile of sEng were more likely to deliver early compared to those in the lowest quartile (HR: 14.96 95% CI: 8.73−25.62, p<0.0001). Natural log transformed sEng correlated positively with log sFlt1 levels (r = 0.87) and inversely with log PlGF levels (r = −0.79) (p<0.0001 for both). Plasma sEng had comparable area under the curve for prediction of adverse outcomes as measurement of sFlt1/PlGF ratio (0.88 {0.81, 0.95} for sEng versus 0.89 {0.83, 0.95} for sFlt1/PlGF ratio, p = 0.74).

**Conclusions:**

In women with suspected preeclampsia presenting prior to 34 weeks of gestation, sEng performs better than standard clinical evaluation in detecting adverse maternal and fetal outcomes occurring within two weeks of presentation. Soluble endoglin was strongly correlated with sFlt1 and PlGF levels, suggesting common pathogenic pathways leading to preeclampsia.

## Introduction

Preeclampsia is a common complication of pregnancy affecting 5−8% of all pregnant women. A clinical diagnosis of preeclampsia is traditionally defined as development of new onset hypertension and proteinuria after 20****weeks of gestation [1]. The current practice for management of patients with a suspicion for preeclampsia involves admission to the hospital for follow-up and management of elevated blood pressures, assessment of 24-hour urine for proteinuria and various blood tests for the evaluation of end-organ damage [2]. In women carrying preterm fetuses, prolongation of pregnancy is of clear benefit, but this is often dangerous to the pregnant woman herself for whom delivery is the only cure; balancing the needs of the fetus and the needs of the pregnant woman, therefore, becomes a significant clinical challenge. Accurate risk stratification is therefore critical in women with preterm preeclampsia for the appropriate management. Furthermore, in a significant percentage of patients who present with atypical signs and symptoms, risk stratification is important to allow for appropriate downstream testing and follow-up. Patients deemed at high risk for developing an adverse outcome need to be hospitalized often with transfer to a tertiary center for an anticipated preterm delivery, given betamethasone to promote fetal lung maturity, and monitored closely for maternal and fetal status to appropriately time delivery. Conversely, iatrogenic preterm delivery needs to be avoided in women at low risk of adverse outcomes.

**Table 1:** Characteristics of all Subjects and Stratified by Adverse Outcome Status.

Variable	All patients	No Adverse Outcomes	Adverse Outcomes	p-value
**N**	170	114	56	
**Baseline**				
Gestational Age (weeks)	31.1 (28.1, 32.9)	30.9 (28.1, 32.9)	31.9 (28.3, 32.9)	0.60
Age (years)	32 (27, 36)	32 (28, 36)	31 (27, 36)	0.37
Body Mass Index (kg/m^2^)	33.0 (29.2, 38.5)	33.7 (30.3, 38.5)	30.8 (28.0, 38.6)	0.13
Nulliparous (%)	55.9 (95)	55.3 (63)	57.1 (32)	0.82
Smoker (%)	8.2 (14)	7.9 (9)	8.9 (5)	0.82
Race				0.50
*White/Caucasian*	56.5 (96)	54.4 (62)	60.7 (34)	
*Black/African American*	20.6 (35)	21.1 (24)	19.6 (11)	
*Asian*	5.9 (10)	5.3 (6)	7.1 (4)	
*Other*	17.0 (29)	19.3 (22)	12.5 (7)	
History of Preeclampsia	21.2 (36)	21.1 (24)	21.4 (12)	0.96
History of Chronic Hypertension	31.8 (54)	33.3 (38)	28.6 (16)	0.53
History of Diabetes	10.0 (17)	11.4 (13)	7.1 (4)	0.38
**Presentation**				
Highest SBP in Triage (mmHg)	140 (130, 150)	135 (124, 144)	149 (141, 160)	<0.0001*
Highest DBP in Triage (mmHg)	87 (77, 96)	84 (74, 92)	96 (86, 103)	<0.0001*
Proteinuria (%, N)	35.3 (60)	19.3 (22)	67.9 (38)	<0.0001*
ALT in Triage (U/L)	17 (12, 27)	16 (12, 23)	20 (13, 40)	0.01*
Creatinine in Triage (µmol/L)	53.0 (44.2, 61.9)	44.2 (44.2, 53.0)	61.9 (44.2, 70.7)	0.0001*
Uric Acid in Triage (µmol/L)	261.7 (220.1,339.0)	243.9 (208.2,285.5)	345.0 (273.6,398.5)	<0.0001*
Platelet Count in Triage (10^9^/L)	253 (205, 291)	262 (227, 296)	234 (166, 274)	0.002*
**Outcomes**				
Any Hypertensive Disorder				
*Chronic HTN*	24.1 (41)	28.1 (32)	16.1 (9)	0.09
*Gestational HTN*	17.1 (29)	23.7 (27)	3.6 (2)	0.001*
*Preeclampsia*	31.8 (54)	8.8 (10)	78.6 (44)	<0.0001*
GA of delivery	35 (32, 37)	37 (35, 38)	32 (28, 33)	<0.0001*
Birth weight	2,550 (1,575, 3,270)	2,958 (2,403, 3,365)	1,460 (850, 1,805)	<0.0001*

SBP =  Systolic Blood Pressure, DBP =  Diastolic Blood Pressure, ALT =  Alanine aminotransaminase, HTN =  Hypertension.

Table shows median (quartile 1, quartile 3) or % (n) where appropriate.

Subjects without a populated endoglin value were eliminated from the current analysis.

* Significant at p<0.05 comparing patients with and without adverse outcomes.

To convert creatinine to mg/dL- divide by 88.4. To convert uric acid to mg/dL- divide by 59.48.

Clinical studies have shown that it is challenging to predict adverse outcomes in women with a suspicion of preeclampsia [3], [4], [5]. In a recent study, von Dadelszen and colleagues used various clinical parameters in patients admitted with a diagnosis of preeclampsia to compute the risk of developing adverse outcome. Their final model included gestational age, serum creatinine, platelet count, aspartate aminotransferase, oxygen saturation and chest pain or dyspnea. The area under curve (AUC) for the prediction of adverse maternal outcomes within 48****hours of study eligibility was 0.88 (95%CI 0.84−0.92) and up to 7****days was >0.7 [6].

**Figure 1:**
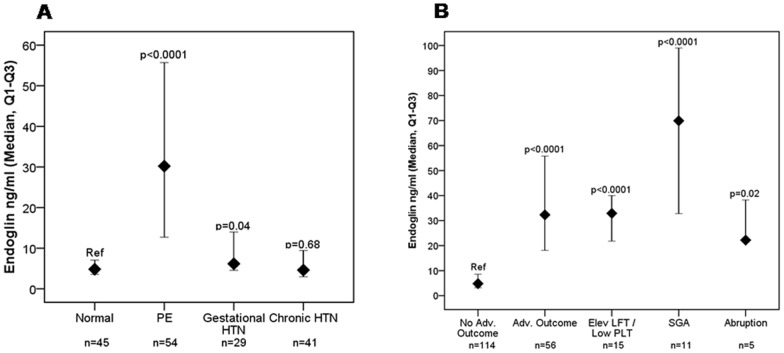
sEng levels among women with suspected preeclampsia. A: Distribution of sEng levels at initial presentation according to diagnosis ascertained at 2 weeks. Median and 25^th^−75^th^ percentile for sEng levels at presentation stratified by diagnosis. All diagnoses were ascertained 2****weeks after presentation. All p values presented on the figure are compared to those without a diagnosis at 2****weeks (“normal”, reference). The median (25th, 75^th^ percentile) of sEng (ng/ml) for each group are as follows: Normal = 4.8 (3.5, 7.1), PE = 30.2 (12.7, 55.7), gestational HTN = 6.2 (4.5, 14.0), chronic HTN = 4.6 (3.0, 9.4). **B: Distribution of sEng levels at initial presentation according to adverse outcomes ascertained at 2 weeks.** Median and 25^th^−75^th^ percentile for sEng levels at presentation stratified by adverse outcomes. All outcomes were ascertained 2****weeks after presentation. All p values presented on the figure are compared to those without an adverse outcome at 2****weeks (“normal”, reference). The median (25th, 75^th^ percentile) of sEng (ng/ml) for each group are as follows: No adverse outcome = 4.8 (3.2, 8.6), adverse outcome = 32.3 (18.1, 55.8), elevated liver function tests/low platelets = 32.9 (21.8, 40.0), small for gestational age (SGA) = 69.9 (32.8, 99.0), abruption = 22.2 (21.8, 38.2).

Data from our laboratory and others have shown that angiogenic factors are altered in women with preeclampsia at the time of clinical diagnosis and weeks to months before the clinical onset of disease [7]. It has been shown that levels of soluble fms-like tyrosine kinase (sFlt1) and soluble endoglin (sEng) are elevated while placental growth factor (PlGF) are reduced in women with diagnosis of preeclampsia and these levels correlate with disease severity and gestational age [8], [9], [10], [11]. The availability of automated assays for sFlt1 and PlGF, has enabled researchers to determine the clinical utility of these markers in the diagnosis and management of preeclampsia [12], [13], [14], [15], [16].

**Figure 2:**
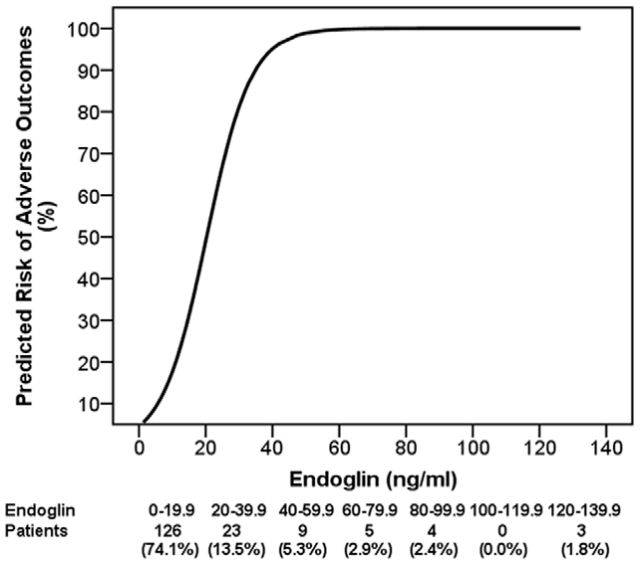
sEng levels and predictive risk of adverse outcome. The cumulative predicted risk of adverse outcomes at different levels of sEng. The sample sizes shown below the figure represent the number of patients at risk for each 20****ng/ml increment of sEng.

While there are number of clinical studies evaluating the role of sFlt1 and PlGF in preeclampsia, there is a paucity of data demonstrating clinical utility for plasma sEng alterations in women with preeclampsia. sEng has also been previously shown to be elevated in women with established preeclampsia, especially in those presenting prematurely and/or in mothers carrying a growth restricted fetus [10], [17], [18]. In a cohort of patients presenting to the obstetrical triage unit prematurely (<34****weeks) that have been previously evaluated for sFlt1 and PlGF [15], we compared sEng with that of standard evaluation in the prediction of adverse maternal and neonatal outcomes occurring within 2****weeks of presentation. We also explored whether the addition of sEng to sFlt1/PlGF ratio improves the prediction of adverse outcomes.

**Table 2:** Detection of Adverse Outcomes in 2 Weeks using various clinical markers and sEng.

FPR	Highest BP	Highest BP + protein	Highest BP + Protein + Platelets + ALT + UA	Endoglin	Highest BP + Protein + Endoglin
2	17.0 (7.4, 26.5)	28.8 (17.3, 40.4)	37.9 (25.4, 50.4)	57.1 (44.2, 70.1)	64.3 (51.7, 76.8)
5	30.5 (18.8, 42.3)	50.9 (38.1, 63.6)	55.2 (42.4, 68.0)	69.6 (57.6, 81.7)	73.2 (61.6, 84.8)
10	32.2 (20.3, 44.1)	61.0 (48.6, 73.5)	63.8 (51.4, 76.2)	80.4 (70.0, 90.8)	76.8 (65.7, 87.8)

FPR =  False Positive Rate.

The detection rate is the percentage of those with adverse outcomes in 2 weeks detected with the use of a given marker based on the FPR and its associated odds ratio cut-off. Confidence intervals were derived from the binomial distribution.

## Methods

### Ethics Statement

The study was approved by the Beth Israel Deaconess Medical Center Institutional Review Board (IRB), and all patients provided written informed consent.

**Figure 3:**
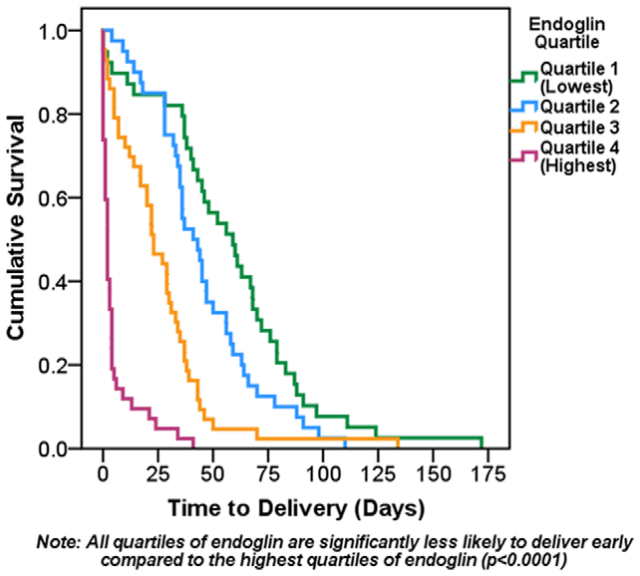
Kaplan-Meier estimates of time to delivery according to sEng quartiles. Kaplan-Meier survival function for time to delivery in all participants by quartile of sEng is depicted. Subjects remained in the analysis until delivery. Univariate Cox Proportional Hazard Models showed quartiles 3 and 4 of sEng had a significantly higher risk of early delivery (HR: 3.14, 95% CI: 1.99−4.98 and HR: 14.96, 95% CI: 8.73−25.62; both p<0.0001) compared to the lowest quartile. The second quartile showed no significant differences in time to delivery compared to quartile 1 (p = 0.07).

### Study Population

This is part of an ongoing prospective cohort study on all pregnant patients who present to obstetrical triage at Beth Israel Deaconess Medical Center (BIDMC), Boston, MA, as described elsewhere [15]. These patients were either referred by their obstetric provider because of signs of preeclampsia or self-presented with symptoms of preeclampsia. Patients were included if the triage care provider deemed an evaluation of preeclampsia necessary. Samples were collected within one hour of arrival to triage and stored at −80°C for analysis. For this current study, all singleton women presenting <34****weeks of gestation and enrolled between July 2009−October 2010 were included, as described elsewhere [15].

**Figure 4:**
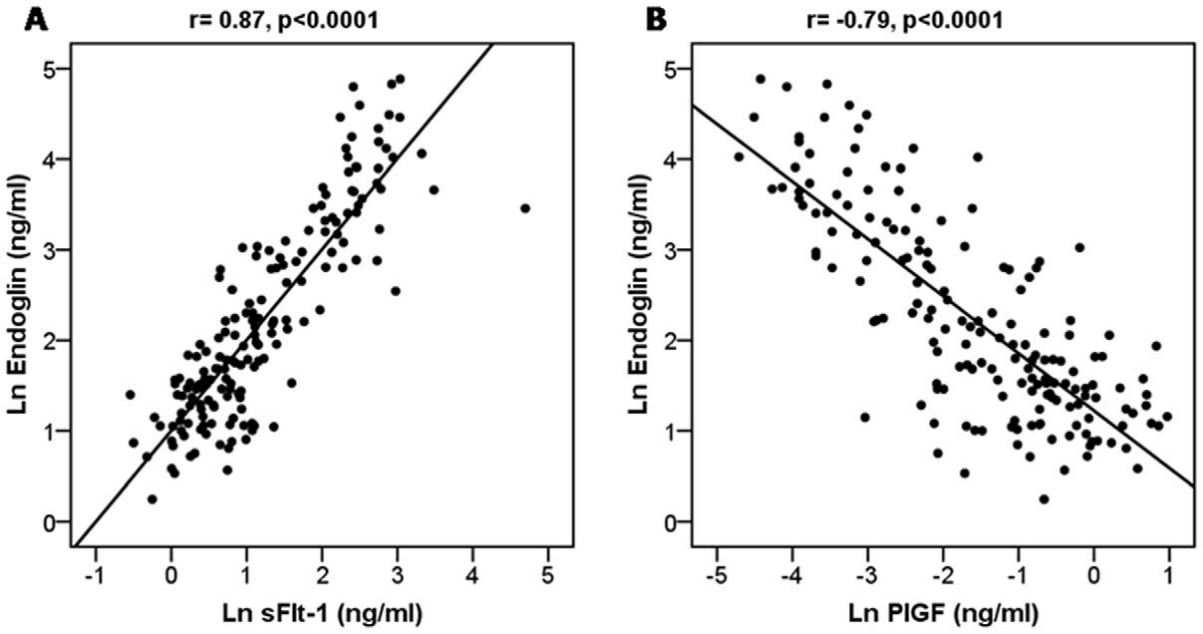
Correlation between sFlt1 and sEng (panel A) and between PlGF and sEng (Panel B). Variables were natural log transformed and p-values were derived from Pearson correlation coefficients.

**Table 3:** Comparison of sEng, PlGF, and sFlt1 among Different Outcomes.

Outcomes	sEng	PlGF	p-value*	sFlt1	p-value**
Adverse Outcome	0.88 (0.81, 0.95)	0.87 (0.80, 0.93)	0.55	0.87 (0.80, 0.93)	0.44
Elev LFT/Low PLT	0.82 (0.73, 0.91)	0.81 (0.69, 0.92)	0.61	0.85 (0.74, 0.96)	0.43
SGA	0.93 (0.88, 0.98)	0.92 (0.88, 0.97)	0.75	0.87 (0.79, 0.95)	0.003*
Abruption	0.70 (0.40, 0.99)	0.76 (0.58, 0.94)	0.41	0.80 (0.67, 0.92)	0.37
DIC	0.85 (0.78, 0.93)	0.75 (0.32, 1.00)	0.61	0.88 (0.63, 1.00)	0.89

* Model performs significantly different at p<0.05 comparing sEng to PIGF.

** Model performs significantly different at p<0.05 comparing sEng to sFlt1.

Elev LFT/Low PLT =  elevated LFT's (aspartate aminotransferase and alanine aminotransferase)/ Low platelets.

SGA =  small for gestational age, DIC =  disseminated intravascular coagulation.

**Table 4:** Comparison of sFlt1/PlGF and combination of sEng among Different Outcomes.

Outcomes	sEng	sFlt1/PlGF	p-value*	sEng+sFlt1/PlGF	p-value**
Adverse Outcome	0.88 (0.81, 0.95)	0.89 (0.83, 0.95)	0.74	0.89 (0.83, 0.95)	0.41
Elev LFT/Low PLT	0.82 (0.73, 0.91)	0.85 (0.73, 0.96)	0.33	0.83 (0.72, 0.94)	0.16
SGA	0.93 (0.88, 0.98)	0.91 (0.86, 0.96)	0.26	0.94 (0.90, 0.98)	0.01*
Abruption	0.70 (0.40, 0.99)	0.79 (0.63, 0.94)	0.32	0.76 (0.56, 0.95)	0.37
DIC	0.85 (0.78, 0.93)	0.91 (0.87, 0.96)	0.10	0.87 (0.73, 1.00)	0.51

* Model performs significantly different at p<0.05 comparing sEng to sFlt1/PIGF.

** Model performs significantly different at p<0.05 comparing sFlt1/PIGF to sEng +sFlt1/PlGF.

Elev LFT/Low PLT =  elevated LFT's (aspartate aminotransferase and alanine aminotransferase)/ Low platelets.

SGA =  small for gestational age, DIC =  disseminated intravascular coagulation.

### Measurement of soluble endoglin (sEng)

Enzyme-linked immunosorbent assay (ELISA) for sEng was performed with commercially available kits, as previously described (R&D systems, Minneapolis, MN). Assays were performed in duplicate and values averaged. The correlation coefficient between duplicate results was 0.97. The intraassay and interassay coefficients of variation were 3.0 and 6.3%. Plasma levels of sFlt1 and PlGF in this same population have been previously published [15].

### Clinical Data

All participants' charts were reviewed to determine outcomes within two weeks of their initial preeclampsia evaluation. We chose a two week interval because previous studies have shown that the levels of angiogenic factors start to rise five weeks before clinical disease and peak two weeks prior to the manifestation of the clinical syndrome [8], [19]. All available clinical data were collected during the two weeks after the initial preeclampsia evaluation including age, race, height, weight, smoking status, gestational age at the time of triage visit, clinical findings, blood pressure (BP), and the results of laboratory tests and fetal scans. All pregnancy outcomes including complications and delivery characteristics such as route of delivery, birth weight and diagnosis of hypertensive disorder were recorded.

### Primary Outcome

The diagnoses of preeclampsia, gestational hypertension, and chronic hypertension were based on American College of Obstetrics and Gynecology (ACOG) criteria with some minor modifications [1]. Preeclampsia was defined as hypertension (a blood pressure ≥140/90 on two occasions 2****hours to 2****weeks apart) after 20****weeks of gestation and proteinuria of ≥300****mg/24****hour or urine protein to creatinine (P/C) ratio of ≥0.3 after 20****weeks of gestation. Gestational hypertension was defined as the presence of hypertension as defined above without proteinuria and chronic hypertension was defined as the presence of hypertension prior to 20****weeks of gestation. We diagnosed superimposed preeclampsia in women with chronic hypertension if there was a significant increase in blood pressure compared with baseline (≥30****mm Hg systolic, ≥15****mm Hg diastolic) in association with new-onset proteinuria (either 300****mg per 24****hours or urine P/C ratio of ≥0.3). If proteinuria was present at baseline, superimposed preeclampsia was diagnosed if there was doubling of urinary protein excretion after 20****weeks' gestation in association with a significant increase in BP. Superimposed preeclampsia was also diagnosed if blood pressure was elevated and there were elevated liver enzymes (two times baseline) or a low platelet count (≤100×10^9^/L).

Adverse maternal outcomes were defined as the presence of hypertension as defined above plus one of the following: elevated liver function test (LFT) including aspartate aminotransferase (AST) or alanine aminotransferase (ALT) (≥80****U/L), thrombocytopenia (platelet count ≤100×10^9^/L), disseminated intravascular coagulation (DIC), abruption (clinical and/or pathological), pulmonary edema, cerebral hemorrhage, seizure (in a woman without an underlying seizure disorder), acute renal failure (creatinine >132.6****µmol/L), or maternal death [20]. The adverse fetal/neonatal outcomes included iatrogenic delivery indicated for hypertensive complications of pregnancy as reported by the primary obstetrician, small for gestational age birth weight (≤10^th^ percentile for gestational age), abnormal umbilical artery Doppler (absent or reverse flow), fetal death, and neonatal death [20]. The presence of adverse outcomes was determined by two study staff members without knowledge of the assay results. Diagnosis and adverse outcomes were ascertained within 2****weeks of presentation.

### Statistical analysis

Baseline characteristics of patients with and without adverse outcomes were compared using the Wilcoxon rank sum and chi-square tests where appropriate. As the distributions of many of the clinical characteristics were highly skewed, baseline measures are presented as medians, 25^th^−75^th^ percentile. The Wilcoxon rank sum test was also used to compare levels of sEng by diagnosis and type of adverse outcome. Pearson correlation coefficients were used to examine the associations between natural log transformed sEng levels and predictors of preeclampsia while Spearman correlations were used to examine the relationship between sEng levels and other baseline factors. Univariate and multivariate logistic regression analysis were used to evaluate significant predictors of adverse outcomes as well as to examine the predicted risk of adverse outcomes at different values of sEng. We computed the detection rate (the percentage of subjects with adverse outcomes within 2****weeks of triage by odds ratio cut-offs) at various false positive rates for sEng as well as various biomarkers. Confidence intervals for the detection rate were estimated using the binomial distribution. To determine the clinical utility of sEng we used receiver operating characteristic (ROC) analysis. To compare areas under the curve we used sFlt1, PIGF, and the sFlt1/PlGF ratio as the reference groups as these markers for adverse outcomes has been established previously. To find the best predictive value of adverse outcomes for various cut-points of sEng, we calculated sensitivity, specificity, positive predictive value (PPV) defined as the proportion of sEng results above the cut-point that are true positives, and negative predictive value (NPV), the proportion of sEng results below the cut-point that are true negatives. Time to delivery by quartiles of sEng levels was visualized using Kaplan Meier curves and calculated using Cox Proportional Hazard Models. Delivery within 2****weeks by quartiles of sEng levels was analyzed using a chi-square test. SAS software, version 9.2 (SAS Institute), was used for all analyses. Two-tailed p-values of <0.05 were considered significant.

## Results

### Demographic and Clinical Characteristics

Demographics of all patients which are part of the original cohort are described elsewhere [15]. During the study period, 815 preeclampsia evaluations took place. One hundred seventy-six evaluations occurred <34****weeks' gestation. Of these evaluations, 6 were eliminated due to missing blood samples. Of the remaining 170 evaluations, 18 (10.6%) were repeat evaluations of women previously enrolled. Baseline characteristics at presentation to obstetric triage of all women and women who did and did not experience subsequent adverse outcomes are shown in **Table 1**.

Over a course of two weeks, adverse outcomes occurred in 32.9% of subjects (N = 56). The most common adverse outcome was indicated delivery (96.4%, N = 54). Of those patients with adverse outcomes, 20% had fetal growth restriction and 27% had HELLP syndrome. Patients who had adverse outcome had higher systolic and diastolic blood pressures (p<0.0001), higher proteinuria (p<0.0001), higher alanine aminotransferase (p = 0.01), higher uric acid (p<0.0001) and lower platelets (p = 0.002) than women who did not experience an adverse outcome. (**Table 1**).

### sEng and subsequent clinical outcomes

The median (25^th^−75^th^ percentile) plasma sEng levels (ng/ml) were significantly higher in women who developed preeclampsia (30.2 {12.7, 55.7}) and gestational HTN (6.2 {4.5, 14.0}) compared with women with no hypertensive disorder (4.8 {3.5, 7.1}; p<0.0001, and p = 0.04 respectively). The levels were similar in patients with chronic hypertension and normal pregnancy outcomes (**Figure 1A** **)**.

Importantly, levels of plasma sEng (ng/ml) were higher in women who experienced any adverse outcome compared to women who did not (32.3 {18.1−55.8} vs 4.8 {3.2−8.6} p<0.0001). In the subgroup analysis, levels of sEng (ng/ml) were higher in women with elevated LFT/low platelet (32.9 {21.8, 40.0}), SGA (69.9 {32.8, 99.0}), and abruption (22.2 {21.8, 38.2}) compared to women with no adverse outcomes (4.8 {3.2, 8.6}; p<0.0001 for elevated LFT/low platelet and SGA and p = 0.02 for abruption). (**Figure 1B**).

### Predictive accuracy of sEng

In univariate logistic regression models greater sEng, systolic blood pressure, diastolic blood pressure, proteinuria, uric acid, creatinine, and lower platelet count were associated with higher risk of adverse outcomes (p<0.001 for sEng, SBP, DBP, proteinuria, and uric acid; p = 0.008 for creatinine; p = 0.006 for platelet count).

On multivariate analysis (controlling for maternal age, parity, BMI, and smoking status), only platelet count (p = 0.047) and sEng (p = 0.02) remained significantly associated with adverse outcomes. To evaluate dose response, we calculated the predictive risk of adverse outcomes by sEng level and noted that higher levels of sEng were associated with greater risk of adverse outcomes (**Figure 2** **)**.

The rates of detection of adverse outcomes using various clinical markers each at different false positive rates are shown in **Table 2**. At each false positive rate, sEng had the highest detection rate compared to blood pressure, proteinuria, platelet count, alanine aminotransaminases and uric acid in different combinations. In addition, hypertension and proteinuria added to sEng did not substantially improve its detection of adverse events at higher false positive rates.

We performed receiver operating characteristic (ROC) analysis and found that AUC for sEng alone was 0.88 (0.81, 0.95) and 0.93 (0.89, 0.97) for sEng combined with blood pressure and proteinuria (p = 0.03). Using a cut-off of 12****ng/ml for sEng a total of 107 (62.9%) subjects fell at or below the cut-off while 63 (37.1%) were above. This cut-off showed high diagnostic accuracy for adverse outcomes with a sensitivity of 80.4%, a specificity of 88.6%, a PPV of 77.6%, a NPV of 90.2%, a positive LR of 7.1 and negative LR of 0.2. Close examination of the false negatives suggested that 8 of 9 subjects that had adverse outcomes with low sEng values had no signs of end-organ damage, but had indicated delivery (**Table S1**). Among the false positives, 12/16 had preeclampsia related delivery ≤37****weeks beyond 2****weeks (average time between triage evaluation and outcome was 5.2****weeks) (**Table S2**). Subjects at or below the cut-off had significantly higher BMI (p = 0.001) and platelet count (p<0.0001) and lower systolic blood pressure (p<0.0001), ALT (p = 0.003), creatinine (p<0.0001), and uric acid (p<0.0001).

### sEng and time to delivery

Kaplan-Meier curves depicting cumulative probability of remaining undelivered for women in the four quartiles for sEng levels are shown in **Figure 3**. The highest sEng levels (4^th^ quartile) were associated with shorter time to delivery. The proportion of patients that delivered within 2****weeks was 90.4% among those patients with sEng levels in the 4th quartile (highest levels), as compared to 14.3% among those with sEng levels in the 1st quartile (lowest levels) (p<0.0001). Those in the highest quartile of sEng were 15****times more likely to deliver early compared to those in the lowest quartile (HR: 14.96 95% CI: 8.73−25.62, p<0.0001) This relationship was slightly attenuated but remained significant after adjustment for gestational age at presentation, highest systolic blood pressure measured in triage, and proteinuria at presentation (HR: 8.10 95% CI: 4.49−14.64, p<0.0001).

### sEng and others angiogenic factors

Natural log transformed levels of sEng were positively correlated with log sFlt1 (r_s_ = 0.87, p<0.0001) and inversely to log PlGF (r_s_ = −0.79, p<0.0001) **Figure 4A and 4B**. Similarly, levels of sEng were found to be strongly correlated with timing of delivery, BP in triage, serum creatinine, and UA (r_s_−0.70, 0.44, 0.34, 0.49 all p<0.0001). sEng showed comparable performance to sFlt1, PlGF and sFlt1/PlGF for the prediction of adverse outcomes. Addition of sEng to sFlt1/PlGF ratio did not improve the sensitivity of the ratio. (**Table 3** **and** **4**).

## Discussion

In this clinical study, we demonstrate that plasma sEng levels were associated with adverse maternal and perinatal outcomes among women presenting to obstetric triage with suspicion of preterm preeclampsia. In particular, sEng was 10-fold elevated in women carrying a growth restricted fetus. Furthermore, levels of sEng were strongly associated with other predictors of preeclampsia-related adverse outcomes similar to sFlt1 and PlGF levels suggesting common pathogenic pathways leading to preeclampsia [15]. sEng performed better than current clinical standards showing high sensitivity and specificity, however it did not improve the sensitivity of other angiogenic biomarkers (sFlt1/PlGF ratio) for prediction of adverse maternal and or fetal outcomes.

Since the conventional clinical parameters lack sufficient sensitivity and specificity [4], [5], it is desirable to identify a biomarker whose levels bear a close relation to the probability of developing adverse outcomes in women with suspicion of preeclampsia. This is akin to the use of troponin for rapid and reliable diagnosis of acute myocardial infarction in patients presenting with chest pain [21], [22]. Our findings in this prospective cohort indicate that, in patients first presenting to obstetric triage elevated sEng levels predict an increased risk of developing preeclampsia related adverse outcomes including indicated preterm delivery. sEng levels provide prognostic information beyond that supplied by demographic characteristics and clinical presentation. Incorporation of sEng in evaluation of these patients may allow early identification of patients at risk for adverse outcomes necessitating timely transfer to a tertiary care center, administration of betamethasone and also potentially reducing unnecessary admissions and intervention.

Our findings extend the observation of benefits of measuring angiogenic factors in patients with suspicion of preeclampsia [15], [17], [23]. In comparison to Chaiworapongsa et****al or Verlohren et****al [17], [23] our study was performed prospectively with analysis of adverse maternal and perinatal outcomes rather than the diagnosis of preeclampsia.

These findings also have important implications for the prediction of preeclampsia. Measurement of antiangiogenic factors early in pregnancy has limited use for prediction of preeclampsia with the exception of early-onset preeclampsia [24], [25]. It has therefore been argued that preeclampsia has “multiple” etiologies. Our previous work [15] and now findings from this article strongly contradicts that view. Thirty years ago Fisher et****al performing clinical pathological correlation (renal biopsies) studies suggested the clinical diagnosis of preeclampsia was erroneous in 15% of nulliparas and nearly 40% of multiparas [26]. Prediction studies, where the diagnosis of preeclampsia was made by hypertension and proteinuria were therefore bound to have poor results whatever marker tested. Our findings when combined with results from animal studies using sFlt1 and sEng to produce preeclampsia phenotypes (including endotheliosis) [27], [28], [29] provide strong evidence for a single entity that correlates with adverse maternal and fetal outcomes. It may therefore be worthwhile to re-define preeclampsia based on a biochemical definition that incorporates angiogenic factors and then use this as a gold standard for the prediction studies.

Several limitations of our study must be acknowledged. The study population was a selected, single center, high-risk group of women with suspicion of preeclampsia. Although approximately 33% percent of the patients developed an adverse outcome within 2****weeks, the most common adverse outcome was indicated delivery. Only a small number of patients developed serious adverse outcomes related to end organ damage such as HELLP syndrome or acute renal failure. Additionally, we could not evaluate rare adverse outcomes such as eclampsia or fetal death because of limited sample size. In our study, we also did not evaluate subjective components such as experience of the physicians, individual attitudes and opinions that might have influenced the decision to delivery. While sFlt1 and PlGF can now be measured on an automated platform [12], the time required for measurement of sEng by manual ELISA limits its value as a diagnostic or prognostic tool for short-term use. Rapid automated assays that quantify the levels of sEng may need to be developed but cutoff values must be established that will yield equally compelling prognostic information.

At preterm gestational ages, safely prolonging pregnancy is associated with significant fetal benefits, therefore it is of utmost importance to find a biomarker that is sensitive and reliable so as not to end pregnancies sooner than necessary. In addition, healthcare costs associated with unnecessary admissions, multiple evaluations and the morbidity related to iatrogenic premature delivery could be reduced by using a test with a high negative predictive value. This study shows that the sEng levels measured in triage is a powerful, independent marker of risk in patients with preeclampsia and related adverse outcomes. Using the current clinical criteria for diagnosing hypertension and proteinuria along with sEng levels may facilitate accurate risk assessment of these patients. Larger studies needs to be done to determine whether use of angiogenic biomarkers in triage will effectively identify women at true risk of adverse outcomes and will allow physicians to safely delay deliveries in patients with favorable angiogenic profiles.

## Supporting Information

Table S1 **Clinical features of participants with sEng <12 (ng/ml) at presentation who experienced adverse outcomes within 2 weeks.**
(DOC)

Table S2 **Clinical features of participants with sEng ≥12 (ng/ml) at presentation, but no adverse outcomes occurring within 2 weeks.**
(DOC)

## References

[1] ACOG practice bulletin (2002) Diagnosis and management of preeclampsia and eclampsia. Obstet Gynecol 99: 159–167. 16175681 10.1016/s0029-7844(01)01747-1

[2] Sibai B, Dekker G, Kupferminc M (2005) Pre-eclampsia. Lancet 365: 785–799. 15733721 10.1016/S0140-6736(05)17987-2

[3] Ganzevoort W, Rep A, de Vries JI, Bonsel GJ, Wolf H (2006) Prediction of maternal complications and adverse infant outcome at admission for temporizing management of early-onset severe hypertensive disorders of pregnancy. Am J Obstet Gynecol 195: 495–503. 16643825 10.1016/j.ajog.2006.02.012

[4] Menzies J, Magee LA, Macnab YC, Ansermino JM, Li J, et al (2007) Current CHS and NHBPEP criteria for severe preeclampsia do not uniformly predict adverse maternal or perinatal outcomes. Hypertens Pregnancy 26: 447–462. 18066963 10.1080/10641950701521742

[5] Thangaratinam S, Gallos ID, Meah N, Usman S, Ismail KM, et al (2011) How accurate are maternal symptoms in predicting impending complications in women with preeclampsia? A systematic review and meta-analysis. Acta Obstet Gynecol Scand 90: 564–573. 21355860 10.1111/j.1600-0412.2011.01111.x

[6] von Dadelszen P, Payne B, Li J, Ansermino JM, Broughton Pipkin F, et al (2011) Prediction of adverse maternal outcomes in pre-eclampsia: development and validation of the fullPIERS model. Lancet 377: 219–227. 21185591 10.1016/S0140-6736(10)61351-7

[7] Powe CE, Levine RJ, Karumanchi SA (2011) Preeclampsia, a disease of the maternal endothelium: the role of antiangiogenic factors and implications for later cardiovascular disease. Circulation 123: 2856–2869. 21690502 10.1161/CIRCULATIONAHA.109.853127 PMC3148781

[8] Levine RJ, Maynard SE, Qian C, Lim KH, England LJ, et al (2004) Circulating angiogenic factors and the risk of preeclampsia. N Engl J Med 350: 672–683. 14764923 10.1056/NEJMoa031884

[9] Chaiworapongsa T, Romero R, Kim YM, Kim GJ, Kim MR, et al (2005) Plasma soluble vascular endothelial growth factor receptor-1 concentration is elevated prior to the clinical diagnosis of pre-eclampsia. J Matern Fetal Neonatal Med 17: 3–18. 15804781 10.1080/14767050400028816

[10] Levine RJ, Lam C, Qian C, Yu KF, Maynard SE, et al (2006) Soluble endoglin and other circulating antiangiogenic factors in preeclampsia. N Engl J Med 355: 992–1005. 16957146 10.1056/NEJMoa055352

[11] Erez O, Romero R, Espinoza J, Fu W, Todem D, et al (2008) The change in concentrations of angiogenic and anti-angiogenic factors in maternal plasma between the first and second trimesters in risk assessment for the subsequent development of preeclampsia and small-for-gestational age. J Matern Fetal Neonatal Med 21: 279–287. 18446652 10.1080/14767050802034545 PMC2846114

[12] Verlohren S, Galindo A, Schlembach D, Zeisler H, Herraiz I, et al.. (2010) An automated method for the determination of the sFlt-1/PIGF ratio in the assessment of preeclampsia. Am J Obstet Gynecol 202: 161 e161−161 e111. 10.1016/j.ajog.2009.09.016 19850276

[13] Schiettecatte J, Russcher H, Anckaert E, Mees M, Leeser B, et al (2010) Multicenter evaluation of the first automated Elecsys sFlt-1 and PlGF assays in normal pregnancies and preeclampsia. Clin Biochem 43: 768–770. 20206155 10.1016/j.clinbiochem.2010.02.010

[14] Sunderji S, Gaziano E, Wothe D, Rogers LC, Sibai B, et al.. (2010) Automated assays for sVEGF R1 and PlGF as an aid in the diagnosis of preterm preeclampsia: a prospective clinical study. Am J Obstet Gynecol 202: 40 e41−47. 10.1016/j.ajog.2009.07.025 19762001

[15] Rana S, Powe CE, Salahuddin S, Verlohren S, Perschel FH, et al (2012) Angiogenic factors and the risk of adverse outcomes in women with suspected preeclampsia. Circulation 125: 911–919. 22261192 10.1161/CIRCULATIONAHA.111.054361 PMC3319742

[16] Thadhani R, Kisner T, Hagmann H, Bossung V, Noack S, et al (2011) Pilot study of extracorporeal removal of soluble fms-like tyrosine kinase 1 in preeclampsia. Circulation 124: 940–950. 21810665 10.1161/CIRCULATIONAHA.111.034793

[17] Chaiworapongsa T, Romero R, Savasan ZA, Kusanovic JP, Ogge G, et al (2011) Maternal plasma concentrations of angiogenic/anti-angiogenic factors are of prognostic value in patients presenting to the obstetrical triage area with the suspicion of preeclampsia. J Matern Fetal Neonatal Med 24: 1187–1207. 21827221 10.3109/14767058.2011.589932 PMC3384532

[18] Kleinrouweler C, Wiegerinck M, Ris-Stalpers C, Bossuyt P, van der Post J, et al (2012) Accuracy of circulating placental growth factor, vascular endothelial growth factor, soluble fms-like tyrosine kinase 1 and soluble endoglin in the prediction of pre-eclampsia: a systematic review and meta-analysis. BJOG 119: 778–787. 22433027 10.1111/j.1471-0528.2012.03311.x

[19] Ohkuchi A, Hirashima C, Matsubara S, Takahashi K, Matsuda Y, et al (2011) Threshold of soluble fms-like tyrosine kinase 1/placental growth factor ratio for the imminent onset of preeclampsia. Hypertension 58: 859–866. 21947468 10.1161/HYPERTENSIONAHA.111.174417

[20] Roberts JM, Myatt L, Spong CY, Thom EA, Hauth JC, et al (2010) Vitamins C and E to prevent complications of pregnancy-associated hypertension. N Engl J Med 362: 1282–1291. 20375405 10.1056/NEJMoa0908056 PMC3039216

[21] Ohman EM, Armstrong PW, Christenson RH, Granger CB, Katus HA, et al (1996) Cardiac troponin T levels for risk stratification in acute myocardial ischemia. GUSTO IIA Investigators. N Engl J Med 335: 1333–1341. 8857016 10.1056/NEJM199610313351801

[22] Reichlin T, Hochholzer W, Bassetti S, Steuer S, Stelzig C, et al (2009) Early diagnosis of myocardial infarction with sensitive cardiac troponin assays. N Engl J Med 361: 858–867. 19710484 10.1056/NEJMoa0900428

[23] Verlohren S, Herraiz I, Lapaire O, Schlembach D, Moertl M, et al.. (2012) The sFlt-1/PlGF ratio in different types of hypertensive pregnancy disorders and its prognostic potential in preeclamptic patients. Am J Obstet Gynecol 206: 58 e51−58. 10.1016/j.ajog.2011.07.037 22000672

[24] Kusanovic JP, Romero R, Chaiworapongsa T, Erez O, Mittal P, et al (2009) A prospective cohort study of the value of maternal plasma concentrations of angiogenic and anti-angiogenic factors in early pregnancy and midtrimester in the identification of patients destined to develop preeclampsia. J Matern Fetal Neonatal Med 22: 1021–1038. 19900040 10.3109/14767050902994754 PMC3427777

[25] Powers RW, Jeyabalan A, Clifton RG, Van Dorsten P, Hauth JC, et al (2010) Soluble fms-Like tyrosine kinase 1 (sFlt1), endoglin and placental growth factor (PlGF) in preeclampsia among high risk pregnancies. PLoS One 5: e13263. 20948996 10.1371/journal.pone.0013263 PMC2952583

[26] Fisher KA, Luger A, Spargo BH, Lindheimer MD (1981) Hypertension in pregnancy: clinical-pathological correlations and remote prognosis. Medicine (Baltimore) 60: 267–276. 7242320

[27] Maynard SE, Min JY, Merchan J, Lim KH, Li J, et al (2003) Excess placental soluble fms-like tyrosine kinase 1 (sFlt1) may contribute to endothelial dysfunction, hypertension, and proteinuria in preeclampsia. J Clin Invest 111: 649–658. 12618519 10.1172/JCI17189 PMC151901

[28] Venkatesha S, Toporsian M, Lam C, Hanai J, Mammoto T, et al (2006) Soluble endoglin contributes to the pathogenesis of preeclampsia. Nat Med 12: 642–649. 16751767 10.1038/nm1429

[29] Lindheimer MD, Kanter D (2010) Interpreting abnormal proteinuria in pregnancy: the need for a more pathophysiological approach. Obstet Gynecol 115: 365–375. 20093912 10.1097/AOG.0b013e3181cb9644

