# Supplementary material for: Plasma Concentrations of Soluble Endoglin versus Standard Evaluation in Patients with Suspected Preeclampsia
Source: PLoS One. 2012 Oct 26;7(10):e48259. doi: 10.1371/journal.pone.0048259 (PMC3482204; doi:10.1371/journal.pone.0048259)
Supplement: Table S1 — Clinical features of participants with sEng <12 (ng/ml) at presentation who experienced adverse outcomes within 2 weeks. (DOC) [file pone.0048259.s001.doc]

**Table S1: Clinical features of participants with sEng <12 (ng/ml) at presentation who experienced adverse outcomes within 2 weeks**

| **#ID** | **Soluble endoglin (ng/ml)** | **GA at presentation** | **GA at delivery** | **Adverse outcome** | **Clinical information** |
| --- | --- | --- | --- | --- | --- |
| 153 | 3.5 | 28.0 | 28.0 | Neonatal death | Chronic abruption and oligohydramnios since 16 weeks, Potter’s sequence |
| 227 | 7.2 | 31.0 | 31.2 | Delivery | NRFHT, CHTN |
| 303 | 3.5 | 33.4 | 35.1 | Delivery | HA, mild PE |
| 399 | 4.4 | 31.1 | 32.5 | Delivery | AFLP |
| 427 | 2.8 | 33.0 | 33.2 | Delivery | Abruption, CHTN, no evidence of abruption on pathology |
| 458 | 2.7 | 29.0 | 29.0 | Delivery | HA, mild PE |
| 598 | 2.4 | 32.0 | 32.4 | Delivery | HA, CHTN |
| 718 | 9.4 | 33.4 | 33.4 | Delivery | NRFHT, CHTN |
| 738 | 4.3 | 26.2 | 26.6 | Delivery | Labor, CHTN, Renal disease |

NRFHT= non- reassuring fetal heart tracing, CHTN= chronic hypertension, HA= headache, PE= preeclampsia, AFLP= acute fatty liver of pregnancy
