# Supplementary material for: Plasma Concentrations of Soluble Endoglin versus Standard Evaluation in Patients with Suspected Preeclampsia
Source: PLoS One. 2012 Oct 26;7(10):e48259. doi: 10.1371/journal.pone.0048259 (PMC3482204; doi:10.1371/journal.pone.0048259)
Supplement: Table S2 — Clinical features of participants with sEng ≥12 (ng/ml) at presentation, but no adverse outcomes occurring within 2 weeks. (DOC) [file pone.0048259.s002.doc]

**Table S2: Clinical features of participants with sEng ≥12 (ng/ml) at presentation, but no adverse outcomes occurring within 2 weeks**

| **#ID** | **Soluble endoglin (ng/ml)** | **GA at presentation** | **GA at delivery** | **Final Diagnosis** |
| --- | --- | --- | --- | --- |
| 32 | 31.8 | 32.0 | 37.6 | GHTN |
| 42 | 35.4 | 23.5 | 27.1 | Severe PE, neonatal death |
| 95 | 24.9 | 29.5 | 32.5 | Severe PE, IUGR, AEDF |
| 238 | 14.2 | 32.2 | 36.2 | PE with appendicitis |
| 332 | 19.9 | 33.2 | 35.5 | Severe PE/Severe HTN |
| 339 | 16.5 | 30.4 | 33.0 | HELLP syndrome |
| 400 | 20.6 | 33.0 | 38.3 | CHTN |
| 401 | 19.6 | 33.5 | 37.5 | Mild PE |
| 476 | 14.8 | 20.5 | 39.6 | IgA nephropathy |
| 610 | 14.0 | 33.6 | 37.0 | Mild PE |
| 628 | 16.6 | 30.0 | 36.1 | Mild PE |
| 638 | 23.9 | 29.3 | 34.2 | HELLP syndrome |
| 680 | 16.3 | 31.4 | 35.5 | GHTN, severe HTN |
| 731 | 16.2 | 31.0 | 36.0 | GTHN, severe HTN |
| 741 | 18.0 | 32.6 | 35.5 | Severe PE/Severe HTN |
| 756 | 17.7 | 30.3 | 35.5 | Severe PE/Severe HTN |

GHTN= gestational hypertension, PE= preeclampsia, IUGR= intrauterine growth retardation, AEDF= absent end diastolic flow, HTN= hypertension, HELLP= hemolysis, elevated liver enzymes and low platelets, CHTN= chronic hypertension
